# Compassionate use of interventions: results of a European Clinical Research Infrastructures Network (ECRIN) survey of ten European countries

**DOI:** 10.1186/1745-6215-11-104

**Published:** 2010-11-12

**Authors:** Kate Whitfield, Karl-Heinz Huemer, Diana Winter, Steffen Thirstrup, Christian Libersa, Béatrice Barraud, Christine Kubiak, Lea Stankovski, Xina Grählert, Gabriele Dreier, Sebastian Geismann, Wolfgang Kuchinke, Anke Strenge-Hesse, Zsuza Temesvari, Gyorgy Blasko, Gabriella Kardos, Timothy O'Brien, Margaret Cooney, Siobhan Gaynor, Arrigo Schieppati, Mariantonia Serrano, Fernando de Andres, Nuria Sanz, Raquel Hernández, Germán Kreis, Charlotte Asker-Hagelberg, Hanna Johansson, Adeeba Asghar, Jean-Marc Husson, Jacques Demotes, Christian Gluud

**Affiliations:** 1 Copenhagen Trial Unit (CTU), Centre for Clinical Intervention Research, Copenhagen University Hospital (DCRIN), Copenhagen, DK-2100, Denmark; 2 Koordinierungszentrum für klinische Studien MUW, Borschkegasse 8b E06, Vienna, 1090, Austria; 3 ATCRIN, Koordinierungszentrum für klinische Studien MUW Borschkegasse 8b E06, Vienna, 1090, Austria; 4 The Danish Medicines Agency, Axel Heides Gade 1, Copenhagen, DK-2300, Denmark; 5 Clinical Investigation Centre CH&U-INSERM, Lille, 9301, France; 6 Institut National de la Santé et de la Recherche Médicale (Inserm), 101 rue de Tolbiac, 75654 Paris, Cedex 13, France; 7 ECRIN Coordination Office, Institut National de la Santé et de la Recherche Médicale (Inserm), 101 rue de Tolbiac, 75654 Paris, Cedex 13, France; 8 Coordinating Centre for Clinical Trials, Medical Faculty - Carl Gustav Carus, University of Technology, Dresden, Germany; 9 ZKS - Clinical Trials Center, University Medical Centre Freiburg, Freiburg, Germany; 10 Coordinating Centre for Clinical Trials, Heinrich-Heine-University, Duesseldorf, Germany; 11 Network of Coordinating Centres for Clinical Trials (KKS Network), University of Cologne, Cologne, Germany; 12 Ministry of Health Social and Family Affairs, Medical Research Council, Budapest, Hungary; 13 Ministry of Health, Medical Research Council, Hungarian ECRIN Committee (HECRIN), Budapest, 1051, Hungary; 14 Molecular Medicine Ireland (MMI), Irish Clinical Research Infrastructure Network (ICRIN) and the National University of Ireland Galway, Newman House 85a St Stephen's Green, Dublin, Ireland; 15 Farmacologiche Mario Negri (IRFMN), Instituto Superiore di Sanita (ISS), Milano, Italy; 16 Spanish Medicines Agency and Medical Devices (AEMPS), Madrid, Spain; 17 Hospital Clinic i Provincial de Barcelona (SCReN), 170 Villarroel, Barcelona, 08036, Spain; 18 Karolinska University Hospital (SweCRIN), Hälsingegatan 43, Stockholm, SE-171 76, Sweden; 19 NIHR Clinical Research Network Coordination Centre, Leeds, UK; 20 European Forum for Good Clinical Practice, Brussels, Belgium

## Abstract

**Background:**

'Compassionate use' programmes allow medicinal products that are not authorised, but are in the development process, to be made available to patients with a severe disease who have no other satisfactory treatment available to them. We sought to understand how such programmes are regulated in ten European Union countries.

**Methods:**

The European Clinical Research Infrastructures Network (ECRIN) conducted a comprehensive survey on clinical research regulatory requirements, including questions on regulations of 'compassionate use' programmes. Ten European countries, covering approximately 70% of the EU population, were included in the survey (Austria, Denmark, France, Germany, Hungary, Ireland, Italy, Spain, Sweden, and the UK).

**Results:**

European Regulation 726/2004/EC is clear on the intentions of 'compassionate use' programmes and aimed to harmonise them in the European Union. The survey reveals that different countries have adopted different requirements and that 'compassionate use' is not interpreted in the same way across Europe. Four of the ten countries surveyed have no formal regulatory system for the programmes. We discuss the need for 'compassionate use' programmes and their regulation where protection of patients is paramount.

**Conclusions:**

'Compassionate use' is a misleading term and should be replaced with 'expanded access'. There is a need for expanded access programmes in order to serve the interests of seriously ill patients who have no other treatment options. To protect these patients, European legislation needs to be more explicit and informative with regard to the regulatory requirements, restrictions, and responsibilities in expanded access programmes.

## Background

'Compassionate use' programmes in Europe allow a medicinal product, without marketing authorisation, to be given to patients with a life-threatening disease when no alternative authorised treatment exists [1]. The goal of 'compassionate use' is to serve the interests of the patient.

European Regulation 726/2004/EC legislates for 'compassionate use' programmes in the European Union [1]. It allows groups of patients with a chronic, seriously debilitating, or life-threatening disease, without a satisfactory authorised treatment available, and who cannot take part in a clinical trial, access to an unlicensed medicinal product [1,2]. The medicinal product concerned must either be the subject of a marketing authorisation application, or under evaluation in a clinical trial.

'Compassionate use' differs from 'off-label' use. In 'off-label' use a licensed medicinal product is prescribed for an indication, or to a patient for which the product is not specifically licensed, whereas, in 'compassionate use' the medicinal product is not licensed and not used as a treatment for any disease. Key differences between 'compassionate use', 'off-label use' and randomised clinical trials are summarised in Table 1. An example of a 'compassionate use' programme is that for the intravenous formulation of Tamiflu (oseltamivir phosphate powder for solution for intravenous infusion) [3,4]. This medicinal product is not licensed but is available to critically ill adults and children with pandemic H1N1 or seasonal influenza A or B infection who did not respond to authorised antivirals or who cannot take Tamiflu orally [3,4].

**Table 1:** Access to medicinal products, through 'compassionate use', 'off-label' use and randomised clinical trials

	'Compassionate use' European regulation	Off-label use	Randomised clinical trial
**Purpose**	Serves the needs of patients where no alternative treatment exists	Serves the needs of patients with an indication other than that the product is marketed for	Serves the needs of society and future patients and may benefit some of the included participants
**Party involved**	Patients	Patients	Participants
**Disease**	A life-threatening or chronically or seriously debilitating disease	Any indication for which the product is not authorised	Any
**Informed consent**	Required in some member states	Not required	Required
**License**	Medicinal product is not yet licensed	Medicinal product is licensed for other indication(s)	Medicinal product can be licensed and not licensed
**Responsible party**	Prescribing physician with approval from the regulatory authorities	Prescribing physician	Sponsor with approval from the regulatory authorities
**Control group**	Without control group	Without control group	With control group
**Data**	In some member states, some data are reported to the regulatory authorities	Spontaneous adverse events may be reported	Outcome measure and adverse event data are reported to the regulatory authorities
**Access to the intervention**	Medicinal product accessed through the programme, afterwards those patients can have access before the product is licensed	Medicinal product available on prescription	Declaration of Helsinki stipulates that participants "are entitled to...share any benefits that result from the trial, for example, access to interventions..."

Regulation 726/2004/EC states that there is a need for a common approach across Europe regarding the criteria and conditions for 'compassionate use' [1]. The European Medicines Agency (EMA) has a role in this harmonisation objective. The EMA's scientific committee, the Committee for Medicinal Products for Human Use (CHMP), can adopt and publish its opinions on the 'compassionate use' programme and the patients targeted [1]. In January 2010 the CMPH published its first opinions on 'compassionate use' programmes in the EU on the EMA website [5]. However, neither Regulation 726/2004/EC nor the CMPH's recommendations are binding on the member states [1].

In order to determine if there is a common approach in Europe, the European Clinical Research Infrastructures Network (ECRIN) surveyed the nature of the national legislation and practice in ten European countries [6], covering approximately 70% of the EU population. Here we describe the results and discuss the impact of 'compassionate use' programmes on clinical practice and clinical intervention research in Europe.

## Methods

The ECRIN working group on regulatory requirements and interaction with competent authorities designed and conducted an international survey [6]. The survey covered national regulations and regulatory procedures for clinical research. The ECRIN working group on regulatory requirements and interaction with competent authorities was composed of two chairpersons, at least one expert from each European ECRIN country, and an ECRIN European correspondent (a person trained in clinical research, working at the national coordination on the implementation of the ECRIN project) from each of following countries: Austria, Denmark, France, Germany, Hungary, Ireland, Italy, Spain, Sweden, and the United Kingdom. The survey was developed in five main phases: drafting, consensus, data collection, validation, and finalising [6].

The two co-chairpersons and two members of the working group on regulatory requirements and interaction with competent authorities drafted the survey. Reaching consensus on the questions in the survey took place during numerous telephone conferences and involved the whole working group. The drafting and consensus process took place between November 2006 and February 2007.

The survey was structured according to category and subcategory of clinical research, with detailed questions for each category (full survey is available in Additional file 1). The survey listed 41 questions, with one question on the specific requirements regarding 'compassionate use' programmes. The format of the survey was an electronic Word document and it was circulated by email to three ECRIN transnational working groups as well as the ECRIN European correspondents in each country (members are listed in the acknowledgements). Members of the working group on regulatory requirements and interaction with competent authorities responded to the survey, additionally, members of the working group on ethics and interaction with ethics committees and members of the working group on adverse event reporting responded to pertinent questions. All answers were collected, discussed, and validated between March 2007 and October 2008. All ten countries surveyed responded to the survey. We did not require ethics approval to perform this questionnaire.

The chairpersons of the working group on regulatory requirements and interaction with competent authorities analysed the completed survey and, where needed, held telephone interviews with the national experts for further information and explanation of specific answers. The results of the survey were discussed and finalised within the working group during numerous teleconferences (2007-2008) and in two face-to-face ECRIN meetings (19-20 May 2007 and 19-20 May 2008). The results of the survey were verified by an informal validation step by representatives from the national competent authorities of the responding countries.

The survey included a section on 'compassionate use' programmes. Here we present the findings relating to 'compassionate use' in ten European countries (Austria, Denmark, France, Germany, Hungary, Ireland, Italy, Spain, Sweden, and the United Kingdom).

The EU Sixth and Seventh Framework Programmes fund the ECRIN project, but had no role in designing the questionnaire, in the collection, analysis, and interpretation of data; in the writing of the report; nor in the decision to submit the paper for publication.

## Results

The European Regulation 726/2004/EC legislates for 'compassionate use' programmes in the European Union. It clearly states that patients must have a chronic, seriously debilitating, or life-threatening disease, that the medicinal product must be undergoing assessment in a clinical trial or be the subject of a marketing authorisation application, and that authorisation of the 'compassionate use' programme itself is necessary [1]. However, Regulation 726/2004/EC lacks details on the authorisation procedures, and ultimately allows the implementation of 'compassionate use' programmes to be governed by individual member states [1].

Results of our survey show that, with the exception of Hungary, all countries surveyed allow for 'compassionate use' programmes. However, there are more differences than similarities in 'compassionate use' programmes in Europe. The single element common to all ten countries was that the responsibility for the 'compassionate use' programme lies with the prescribing physician. Four countries (Hungary, Ireland, Sweden, and the UK) are currently without formal regulatory systems, and for those with national legislation on 'compassionate use' programmes it is varied in both content and comprehensiveness (Table 2). Six of the ten countries surveyed allow 'compassionate use' programmes on a 'named/individual patient' basis (Austria, Denmark, France, Italy, Spain, and the UK), only three countries (Austria, France, and Spain) specify that 'compassionate use' programmes must be outside clinical trials, the opinion of the ethics committee is only sought in two countries (Italy and Spain (and in some UK hospitals)). The contents and requirements of the application for authorisation varies in all the countries surveyed and in most countries the outcomes of the 'compassionate use' programme do not need to be reported to the regulatory authorities. Differing interpretations and regulatory requirements result from Regulation 726/2004/EC not being explicit and because the Regulation allows individual member states to govern the programmes nationally.

**Table 2:** Summary of 'compassionate use' regulations in ten European countries

Country	'Compassionate use'	Responsibility	Authorising agency	Reporting
**Austria**	Termed 'Named patient use' Treatment of individuals Separate from clinical trials	Treating physician	N/A	No
**Denmark**	Termed 'Compassionate use permit' Treatment of individuals Consent to disclose health data required	Treating physician	Danish Medicines Agency (DMA)	Adverse events reported to the DMA
**France**	Termed 'Temporary authorisation for use' for individuals, or 'Cohort temporary authorisation for use' Separate from clinical trials	For 'nominative' use the prescribing physician, for 'cohort' use the license holder	Agence Française de sécurité sanitaire des produits de santé (Afssaps)	All adverse reactions. Periodic report for 'temporary authorisation for use' programmes.
**Germany**	National legislation and guidelines Informed consent required	'Responsible person'	Bundesinstitut für Arzneimittel und Medizinprodukte (BfArM) or Paul-Ehrlich Institut (PEI)	Serious adverse events reported to authorising agency within 15 days
**Hungary**	No specific legislation	N/A	N/A	N/A
**Ireland**	The product must be between a phase III trial and marketing authorisation Guidelines	Prescribing physician	Irish Medicines Board (IMB)	No
**Italy**	Termed 'Compassionate use' for individuals Informed consent required	Treating physician	Ethics committee	No
**Spain**	Termed 'Compassionate use' for individuals Informed consent required Separate from clinical trials	Treating physician	Agencia Española de medicamentos y productes sanitarios (AEMPS)	Efficacy and adverse events reported to AEMPS
**Sweden**	Guidelines	N/A	Medical product agency (MPA)	N/A
**UK**	Termed 'compassionate use' or 'expanded access' using 'specials' for individual patients Guidelines	Prescribing physician	Medicines and Healthcare products Regulatory Agency (MHRA)	Serious adverse reactions reported to MHRA

### National legislation and practice

In Austria, national regulations for 'compassionate use' for groups of patients, in accordance with the European Regulation 2004/726/EC, are currently under preparation. As an alternative, 'named patient use' could be utilised [7]. 'Named patient use' allows a physician to give a patient with a severe condition a medicinal product which has no market approval in Austria. The treating physician has full responsibility. This can only be done on an individual basis, ie, the name of the patient must be known [7]. 'Named patient use' cannot be used in a clinical trial with anonymous patients.

In Denmark, it is possible to carry out 'compassionate use' studies. 'Compassionate use permits' are only permitted for a specific treatment for an individual patient [8]. In special cases the Danish Medicines Agency can authorise the dispensing or sale of a medicinal product, eg, for life threatening diseases for which there are no well-documented treatment options. The treating doctor applies for a 'compassionate use permit' from the Danish Medicines Agency. This is an application to dispense non-authorised medicinal products. The application includes the description of treatment, the expected duration of treatment, product information, scientific literature or other information about effects, adverse reactions, copy of medical records and information about planned monitoring of the course of the disease [8]. If accepted, the applicant receives authorisation. The applicant must notify the pharmacy and include a copy of the authorisation with the prescription. The treating physician is obliged to report back to the Danish Medicines Agency. Adverse reactions related to the medicinal product must be reported to the Danish Medicines Agency. However, the amount of reporting has been reduced to accommodate an increase in requests for 'compassionate use permits', eg, age and sex of the patient no longer need to be reported [8].

In France, use of medicinal products which do not have marketing authorisation and which is outside the context of a clinical trial is dependent on prior 'temporary authorisation for use' (ATU) being granted by the French Health Products Agency (Afssaps) [9,10]. ATU permits are granted as a discretionary, exceptional, and temporary measure, when the following conditions are met: the treatment, prevention, or diagnosis of serious or rare diseases; an absence of a suitable therapeutic alternative (medicinal product or other) available in France and when the benefit/risk ratio of the medicinal product is presumed to be positive. The use of these medicinal products is authorised by Afssaps, for a limited period of time. In practice, there are two types of temporary authorisations for use. Firstly, the 'nominative temporary authorisation for use', issued for a named patient, at the request of and under the responsibility of the prescribing physician. This type of 'temporary authorisation for use' concerns medicinal products of which the efficacy/safety ratio is presumed to be favourable in the light of the data available. Secondly, the 'cohort temporary authorisation for use', which concerns a group or sub-group of participants, treated and monitored according to criteria fully defined in a protocol for therapeutic use and information collection. A 'cohort temporary authorisation for use' is issued at the request of the holder of the licensing rights, who commits to submit a marketing authorisation application within a determined time limit.

In Germany, 'compassionate use' programmes for medicinal products were introduced into legislation with the 14th amendment of the German Medicines Act (AMG) and was updated by the 15th amendment effective from July 2009 [11]. Section 21(2) of the AMG states that under the provisions in Article 83 of European Regulation 726/2004 EC medicinal products which are made available to patients with a disease which leads to severe disability or which is life-threatening and who cannot be satisfactorily treated with an approved medicinal product, do not require marketing authorisation [11]. The 15th amendment also stipulates that dispensing of the medicinal products in 'compassionate use' programmes must be free of charge and exempts these products from the prescription medicine pharmacy chain of distribution [11]. In July 2010, a new ordinance set out precise regulations regarding the duty of the responsible person to notify the competent authority, the need to secure approval from the competent authority, patient informed consent, reporting serious adverse events to the competent authority, and public availability of information about the main characteristics of the programmes [12].

In Hungary there is neither regulation nor implementation of 'compassionate use' programmes.

In Ireland, currently there is no system regulating 'compassionate use' programmes, however, 'compassionate use' programmes can fall under the Irish clinical trial regulations SI 190 of 2004 or SI 540 of 2007 [13]. Clinical trial regulations SI 190 of 2004 require all investigational medicinal product studies to be authorised by the Irish competent authority prior to the start of the trial. SI 540 of 2007 Schedule I, point 5, paragraph 2 exempts a product without a marketing authorisation in Ireland from being imported, however, it can be prescribed by a medical doctor, but responsibility of the oversight of the product is that of the prescriber (medical doctor) [13]. The Irish competent authority has established a statutory notification system for use of unauthorised medicines. It is the responsibility of the wholesaler and manufacturer to notify the Irish competent authority if they receive unauthorised medicines [14].

In Italy, the 'compassionate use' of a medicinal product used in non-authorised conditions in a single patient in exceptional circumstances is allowed and is regulated by the Ministry of Health Decree May 8, 2003 and by the Legislative Decree April 24, 2006 n. 219 [15,16]. The request for 'compassionate use' programmes should be made by the physician who assumes responsibility of administration of the product to the patient. An authorisation should be requested from the ethics committee, and a special informed consent should be prepared.

In Spain, 'compassionate use' is defined as the prescription of a medicinal product used in a non-authorised condition in isolated patients outside the context of a clinical trial, and under the physician's responsibility. An informed consent, a clinical report, a centre authorisation, and the Spanish Agency for Medicines and Medical Devices (Agencia española de medicamentos y productos sanitarios, AEMPS) authorisation are required. The physician should notify the results and adverse reactions to the AEMPS. 'Compassionate use' programmes will be allowed in the period between the application for approval and the decision on market authorisation of the medicinal product [17].

In Sweden, there is no system regulating 'compassionate use' programmes. In general, only commercial sponsors can offer 'compassionate use' programmes and the Swedish Medical Products Agency provisions explain in what situation this is possible. Instead it may be possible to prescribe the study drug after discontinuation of study on a participant-by-participant basis.

In the UK, in the case of clinical trials involving medicinal products or medical devices, the treatment should be extended after the end of the trial if the participant is benefitting fro the product or device, this is known as 'expanded access'. Outside clinical trials and subject to certain conditions, unlicensed medicinal products ('specials') can be manufactured and supplied to individual patients in order to meet the needs of some patients who cannot be treated with licensed medicinal products [18]. The product is for use by individual patients on the prescriber's direct personal responsibility, essential records must be kept, and serious adverse drug reactions reported to the Medicines and Healthcare products Regulatory Agency (MHRA) [18]. The MHRA has issued a guideline on unlicensed medicinal products for individual patients [19]. Ethical review of 'compassionate use' is available in some UK healthcare trusts through a 'clinical ethics committee', but this is not nationwide [20]. A regional body of the UK National Health Service, The Kent and Medway Area Prescribing Committee, has issued additional guidelines for access to unlicensed investigational drugs outside of clinical trials. The guidance includes that the prescriber believes that the risk/benefit profile of the new drug is likely to be favourable to the patient, the prescriber has explained to the patient or carer that the medicine is unlicensed, and the prescriber obtains and documents the patient's consent to treatment before prescribing [21].

## Discussion

Under the European Regulation 726/2004/EC legislation for 'compassionate use' programmes, eligible patients can be granted access to a medicinal product, which is not licensed [1]. One aim of Regulation 726/2004/EC is to foster a common approach to 'compassionate use' programmes across Europe. In order to understand how 'compassionate use' programmes are regulated, and to ascertain if the desired common approach is being achieved in Europe, ECRIN surveyed ten European countries representing approximately 70% of the EU population [6]. We found significant differences in the national regulations for 'compassionate use' programmes.

Through performing the survey and examining the pertinent legislation, it is clear that 'compassionate use' is not a suitable term for these programmes. 'Compassion' describes the wish to relieve suffering, a fundamental principle throughout healthcare. Accessing medicinal products with little knowledge of their benefit or harm should not be labelled as the most compassionate strategy. Relief of suffering is not always achieved through intervening and certainly does not come through causing more harm than good. We therefore prefer the term 'expanded access', as used in the USA [22]. It describes a key element of the programme, i.e., that a medicinal product is made more widely available before it has obtained market authorisation. We will use the term expanded access for the rest of the discussion.

The major weakness of this survey is that the results from 10 member states may not adequately reflect the situation across Europe. However, nine of the ten countries we surveyed do have national legislation and requirements for expanded access programmes; this strong trend may be indicative of the situation in other European countries. For example, Switzerland, although not bound by the EU Regulation, does have legislation for national expanded access programmes [23]. The national competent authority (Swissmedic-Swiss Agency for Therapeutic Products) authorises the programmes under the conditions that the disease is life threatening, the programme is compatible with the protection of health, a significant therapeutic benefit is expected and no comparable medicine exists [23]. Reporting adverse events to Swissmedic is mandatory [24]. The Swiss requirements illustrate that the results of our survey may be quite representative of the European picture.

### European legislation - let's go further

The results show that there are more differences than similarities in expanded access programmes in Europe; it appears that the desired common approach is lacking. There are inherent challenges in striving for harmonisation in 27 member sates, each with differing health care systems, however, the differences in the availability of expanded access programmes and in the protection of the patient remain in part because Regulation 726/2004/EC allows national expanded access programmes to be governed by individual member state legislation [1]. This undermines the expectations of European patients and citizens.

Although Regulation 726/2004/EC clearly states that authorisation of expanded access programmes is necessary, it does not describe any aspects of the required content for the authorisation application nor the authorisation process, and the responsibilities of the prescribing physician, national competent authorities, and the product manufacturer are ambiguous. To achieve a common approach, European legislation needs to be more explicit and rule that: authorisation requires evidence from a randomised clinical trial of greater benefit than harm; competent authorities assess the intervention and independent ethics committees assess the risk to the patient (as is practised in Spain and in some UK hospitals); authorisation is independent of the product manufacturer; an open-access list of authorised programmes is mandatory; safety and efficacy data are reported to the regulatory authorities (as is practised in Denmark, France, Germany, and Spain); depositing the results of the programmes in an open-access database is necessary; and patient information with full informed consent procedures is required. Improved legislation must then apply to all medicinal interventions including surgery and medical devices and be binding on all member states.

The Food and Drug Administration (FDA) has recently implemented improvements, similar to those we present here, to the US regulations on expanded access which include the need for informed consent, assessment by an independent review board, and the reporting of patient outcomes and adverse effects to the FDA [22].

A public register of the CMPH's opinions of specific expanded access programmes on the EMA website was launched in January 2010 and is a positive step towards developing a common approach in Europe [5].

The basis of these additional requirements is to better serve and protect patients, although it is possible that stricter requirements could lengthen the waiting time for access to new interventions. On the opposite, sharing information between the regulatory authorities and the EMA on authorisations and safety and efficacy records should accelerate decision making and access.

### Expanded access cannot replace clinical trials

Expanded access programmes do not reliably inform us of the benefits and harms of an intervention and cannot replace randomised clinical trials (Table 1) [25,26]. A randomised clinical trial tests a medical intervention against a control, the purpose of which is to improve healthcare for society and future patients. Blurring the lines between expanded access and clinical trials risks that expanded access programmes are undertaken as an easy way to collect information on new medicinal products instead of conducting randomised clinical trials. When this happens, the safeguards inherent to clinical trials, eg, having a control group, securing insurance to protect the patients, reporting all adverse events, reporting the results, etc. are all circumvented.

Regulation 726/2004/EC separates expanded access and clinical trials, but many member states do not. Only the Austrian, French, and Spanish national legislation clearly states that expanded access programmes must be conducted separately from clinical trials. Expanded access programmes and clinical trials must be separated, firstly to protect patients from exploitation, and secondly so that both the needs of the seriously ill and the needs of society can be best served.

There are other situations where patients can access unlicensed medicinal products: phase I study participants and clinical trial participants. Some types of phase I studies enrol patients with a serious or terminal disease without treatment options (principally cancer). Although the principal purpose of phase I studies is to assess the safety of the medicinal product, the participants of phase I studies are afforded greater protection than those in the expanded access programme through the regulatory requirements needed for any clinical trial (eg, ethical review, informed consent, insurance). For any type of clinical trial, according to the Declaration of Helsinki, when trial participants benefit from a clinical trial intervention, they should have access to that intervention after the trial has finished [27]. Furthermore, if the intervention is potentially beneficial to patients outside the clinical trial with a chronic, seriously debilitating, or life-threatening disease, without a satisfactory authorised treatment available, then this intervention should be made available to them through an expanded access programme.

### Expanded access is for patients

The purpose of expanded access is to serve the needs of patients. For patients with serious and life-threatening diseases without sufficient treatment options there is an absolute need for expanded access programmes. However, expanded access involves unknown risks. Patients have the right to make informed judgements about taking part in an expanded access programme, just as clinical trial participants do. This must involve informed consent procedures where all options, including that of palliative care or no treatment, are presented objectively, and for those who cannot give their own consent, surrogate consent should be possible. The newly available information from the EMA website is open-access and contributes to informing patients about expanded access programmes in general and about specific programmes [5]. It is paramount that informed consent procedures are implemented in Europe.

## Conclusions

'Compassionate use' is a misleading term and should be replaced with 'expanded access'. To protect patients, European legislation needs to be more explicit and informative with regard to the regulatory requirements, restrictions, and responsibilities in expanded access programmes. With increasing demand from patients [25,26] expanded access programmes must not be left in a grey-legislative area as they concern some of the most vulnerable in society.

## Competing interests

Jean-Marc Husson declares that he has worked for Eudipharm. All other authors declare that they have no competing interests.

## Authors' contributions

CK, CG, and JD designed the survey. All authors contributed equally to the survey responses. All the authors have drafted the manuscript and read, and approved the final version.

## Supplementary Material

Additional file 1 **Copy of the full ECRIN questionnaire on regulatory requirements in clinical research**. Click here for file
