# Supplementary material for: Compassionate use of interventions: results of a European Clinical Research Infrastructures Network (ECRIN) survey of ten European countries
Source: Trials. 2010 Nov 12;11:104. doi: 10.1186/1745-6215-11-104 (PMC2997627; doi:10.1186/1745-6215-11-104)
Supplement: Additional file 1 — Copy of the full ECRIN questionnaire on regulatory requirements in clinical research. [file 1745-6215-11-104-S1.PDF]

ECRIN WP1-WP2-WP3 - 2007 Survey

The ECRIN Working Party 2 focuses on regulatory affairs and interaction with competent authorities. Its first task is to delineate the relevant categories of clinical research as presently defined by national laws, considering that there is no European law on "Clinical/Biomedical Research" as a whole, but also to identify what is required in each country for each type of clinical research. This work may also become useful for the other ECRIN Working Parties – especially WP1 and WP2.

For this purpose we kindly ask you to fill in, as comprehensively as possible, the questionnaire below (except the grey area) and send it before **9th March 2007** to Christine Kubiak at kubiak@tolbiac.inserm.fr. For each of the following categories of clinical research please provide information on national regulation, rules, and practices that a 'sponsor' or a 'sponsor-investigator' would face. We know that we put a lot of questions, but many may be replied by 'copy and paste'. We also know that it requires a lot of knowledge to answer all the questions correctly. This is not a test to your present knowledge, but rather our try to get the most correct information from your country. Therefore, please involve as many experts in your country's guidelines, laws, and practices as you like. We ask primarily the WP1 members to answer the questions pertaining to ethical issues, primarily the WP2 members to answer the questions pertaining to legislation, and primarily the WP3 members of to answer the questions pertaining to adverse events.

Please add a row if any other relevant category (eg, prevention trials, screening trials, quality of life, etc) exists in your country and cannot be described following the proposed frame. Please answer "not a specific category" if a category is not relevant in your country.

Thank you very much for your collaboration.

On behalf of the members of ECRIN WP2,

Very best wishes,

CK, JDM, CG

GLOSSARY

Biomarkers: a characteristic that is objectively measured and evaluated as an indicator of normal biological processes, pathogenic processes or pharmacologic responses to a therapeutic intervention (www.cdisc.org**)**

Surrogate marker: assessment of a drug's biological activity that substitutes for a clinical end point such a death or pain relief. (www.cdisc.org)

Clinical research: biomedical research conducted on human participants

Clinical trial: any investigation in human subjects intended to discover or verify the clinical pharmacological and/or other pharmacodynamic effects of one or more investigational medicinal product(s) , and/or identify any adverse reactions to one or more investigational medicinal product(s), and /or to study the absorption, distribution, metabolism and excretion of one or more investigational medicinal product(s) with the objective of ascertaining its (their) safety and/or efficacy [Directive 2001/20/EC]

Complementary and alternative medicine: is a group of diverse medical and health care systems, practices, and products that are not presently considered to be part of conventional medicine. Complementary medicine is used together with conventional medicine. Alternative medicine is used in place of conventional medicine. (www.nih.gov)

Investigational medicinal product (**IMP**): a pharmaceutical form of an active substance or placebo being tested, or used as a reference in a clinical trial, including products already with a marketing authorisation but used or assembled (formulated or packaged) in a way different from the authorised or when used for an unauthorised indication or when used to gain further information about the authorised form [Directive 2001/20/EC art 2 (d)]

Phase I (most typical kind of study: Human pharmacology)
Studies that assess tolerance, define/describe the pharmacokinetics and pharmacodynamics, explore drug metabolism and drug interactions and estimate activity [ICH E8]

Phase II (most typical kind of study: Therapeutic exploratory)
Studies that explore use for targeted indication, estimate dosage for subsequent studies, provide basis for confirmatory study design, endpoints, methodologies [ICH E8].

Phase III (most typical kind of study: Therapeutic confirmatory)
Studies that demonstrate/confirm efficacy, establish safety profile, provide an adequate basis for assessing the benefit/risk relationship to support licensing, establish dose-response relationship [ICH E8].

Phase IV (variety of studies: Therapeutic use)
Phase IV begins after drug approval.
Studies that refine understanding of benefit/risk relationship in general or special population and/or environments identify less common adverse reactions, refine dosing recommendation [ICH E8].

Vulnerable PARTICIPANTS: Individuals whose willingness to volunteer in a clinical trial may be unduly influenced by the expectation, whether justified or not, of benefits associated with participation, or of a retaliatory response from senior members of a hierarchy in case of refusal to participate. Examples are members of a group with a hierarchical structure, such as medical, pharmacy, dental and nursing students, subordinate hospital and laboratory personnel, employees of the pharmaceutical industry, members of the armed forces, and persons kept in detention.
Other vulnerable **PARTICIPANTS** include patients with incurable diseases, persons in nursing homes, unemployed or impoverished persons, patients in emergency situations, ethnic minority groups, homeless persons, nomads, refugees, minors, and those incapable of giving consent. [ICH]

COUNTRY:			Is a submission to an ethics committee required? (specify the name of the committee and who is responsible for the submission)	Is a submission to competent authority required? (specify the name of the competent authority and who is responsible for the submission)	Is there a specific procedure for substantial amendments?	Is there a requirement for a sponsor in this type of trial? Is co-sponsorship allowed?	Is insurance required? (specify who is covered: sponsor, investigator, patients)	Adverse event (AE) reporting Specify which adverse events have to be reported by the sponsor (or, if no sponsor, by the investigator) when and to whom? Is a safety report requested?	
								Serious adverse events	Non-serious adverse events
**1-CLINICAL TRIALS ON MEDICINAL PRODUCTS j**									
**Phase I**									
**Phase II**									
**Phase III**									
**Phase IV**									
**Specific interventions ,**									
**Biotherapy**	**Tissue engineering**								
	**Cell therapy**								
	**Gene therapy**								
**Biopharmaceuticals**	**Blood-derived products**								
	**Monoclonal antibodies/ recombinant proteins/peptides**								
	**Oligonucleotides**								
**Vaccines ƒ**									
**Fixed combination of medicinal products**									
**Multimodal trials „**									

j Pharmaco-epidemiology is on Section 7 'Epidemiology'
, The use of phase I to IV also applies to these specific interventions. Please specify if there are any particularities for these phases.
ƒ If there are specific requirements for living or attenuated vaccines please specify.
„ A multimodal-therapy trial evaluates the effect of medicinal product together with other medical intervention such as radiotherapy, surgery, etc.

COUNTRY:		Is a submission to an ethics committee required? (specify the name of the committee and who is responsible for the submission)	Is a submission to competent authority required? (specify the name of the competent authority and who is responsible for the submission)	Is there a specific procedure for substantial amendments?	Is there a requirement for a sponsor in this type of trial? Is co-sponsorship allowed?	Is insurance required? (specify who is covered: sponsor, investigator, patients)	Adverse event (AE) reporting Specify which adverse events have to be reported by the sponsor (or, if no sponsor, by the investigator) when and to whom? Is a safety report requested?	
							Serious adverse events	Non serious adverse events
**2- CLINICAL RESEARCH ON MEDICAL DEVICE**								
**Device alone**	**Authorised**							
	**Non-authorised …**							
**Device combined with medicinal products †**	**Authorised**							
	**Non-authorised**							
**3- OTHER THERAPEUTIC TRIALS**								
**Radiotherapy trials**								
**Surgery trials**								
**Transplantation**								
**Transfusion**								
**Physical therapy**								
**Psychotherapy (without medicinal product)**								

… Either non CE labelled or used in another indication.
† Examples: medical device for drug delivery or drug-coated stent

COUNTRY:		Is a submission to an ethics committee required? (specify the name of the committee and who is responsible for the submission)	Is a submission to competent authority required? (specify the name of the competent authority and who is responsible for the submission)	Is there a specific procedure for substantial amendments?	Is there a requirement for a sponsor in this type of trial? Is co-sponsorship allowed?	Is insurance required? (specify who is covered: sponsor, investigator, patients)	Adverse event (AE) reporting Specify which adverse events have to be reported by the sponsor (or, if no sponsor, by the investigator) when and to whom? Is a safety report requested?	
							Serious adverse events	Non-serious adverse events
**4.-DIAGNOSTIC STUDIES**								
**Diagnostic studies (without medicinal product or medical device)**	***In vivo***							
	In vitro							
**Imaging studies (without medicinal product or medical device)**								
**5- CLINICAL RESEARCH ON NUTRITION ‡**								
**Comments**								
**Nutritional studies**								
**Nutritional supplements**								

‡ If necessary please comment on clinical research on nutrition and the border with clinical research on medicinal products

COUNTRY:		Is a submission to an ethics committee required? (specify the name of the committee and who is responsible for the submission)	Is a submission to competent authority required? (specify the name of the competent authority and who is responsible for the submission)	Is there a specific procedure for substantial amendments?	Is there a requirement for a sponsor in this type of trial?<br>Is co-sponsorship allowed?	Is insurance required? (specify who is covered: sponsor, investigator, patients)	Adverse event (AE) reporting<br>Specify which adverse events have to be reported by the sponsor (or, if no sponsor, by the investigator) when and to whom?<br>Is a safety report requested?	
							Serious adverse events	Non-serious adverse events
**6- OTHER CLINICAL RESEARCH**								
Complementary and alternative medicine								
Cosmetics								
Tattoo								
Biobanks: collection of blood, other fluids or tissue samples								
Physiology								
Physiopathology								
Psychology								

COUNTRY:		Is a submission to an ethics committee required? (specify the name of the committee and who is responsible for the submission)	Is a submission to competent authority required? (specify the name of the competent authority and who is responsible for the submission)	Is there a specific procedure for substantial amendments?	Is there a requirement for a sponsor in this type of trial? Is co-sponsorship allowed?	Is insurance required? (specify who is covered: sponsor, investigator, patients)	Adverse event (AE) reporting Specify which adverse events have to be reported by the sponsor (or, if no sponsor, by the investigator) when and to whom? Is a safety report requested?	
							Serious adverse events	Non-serious adverse events
**7- EPIDEMIOLOGY ˆ**								
**Comments**								
**Pharmaco-epidemiology**	**Interventional ‰**							
	**Non-interventional ‰**							
**Epidemiology**	**Interventional ‰**							
	**Non-interventional ‰**							
**Registries of patients (databases) Š**								

ˆ Please give a definition

‰ For the definition, please refer to next page, first question.

Š Information system designed for the collection, storage, management and analysis of data on persons with the same drug, disease or symptoms in a given geographic area. The process is a continual and systematic collection of data.

Is there a definition for interventional vs. non-interventional (or observational) clinical research? ☐yes ☐no

If yes, please specify which of the categories above are considered either observational, or interventional, or not covered by this definition (please give source/reference and if possible add link):

Are studies on usual care / quality studies / clinical audits considered as a specific category? ☐ yes ☐ no

If yes, please specify, give source/reference and if possible add link:

Is there a definition for non-commercial trials? ☐yes ☐no

If yes, please specify, give source/reference and if possible add link:

Is there a definition for a non-commercial sponsor? ☐ yes ☐ no

If yes, please specify, give source/reference and if possible add link:

<u>What is the definition of investigational medicinal products (IMP) in your country?</u> (you can tick more than one box)

☐ Study drug
☐ Comparator
☐ Background treatment (if collecting information on it is one of the objectives of the study)
☐ Background treatment (when the objective of the study is not to gain further information on it)
☐ Challenge drug
☐ Rescue drug
☐ Drug used to assess outcome measure (contrast / imaging, etc...).
☐ Other, please define:

(please give source/reference and if possible add link):

<u>Are there specific requirements for IMP labelling in trials on medicinal products?</u> ☐**yes** ☐**no**
If yes, please specify, give source/reference and if possible add link:

<u>Are there specific requirements for IMP labelling in non-commercial trials?</u> ☐ **yes** ☐ **no**

If yes, please specify, give source/reference and if possible add link:

<u>In non-commercial trials, is there a waiver for the sponsor to purchase the IMP?</u> ☐ **yes** ☐ **no**

If yes, which organisation pays for the IMP? (please give source/reference and if possible add link):

Are there specific requirements regarding compassionate USE studies / compassionate use? ☐yes ☐no

If yes, please specify, give source/reference and if possible add link:

Are there any additional requirements for studies on biopharmaceuticals (proteins, monoclonals, DNA..) ? ☐yes ☐ no

If yes, please specify, give source/reference and if possible add link:

Are there any additional requirements for studies on biotherapy (gene-cell-tissue) ? ☐yes ☐ no

If yes, please specify, give source/reference and if possible add link:

Are there specific requirements for studies using adult stem cells? ☐yes ☐no

If yes, please specify, give source/reference and if possible add link:

Are there specific requirements for studies using embryonic stem cells? ☐yes ☐no

If yes, please specify, give source/reference and if possible add link:

Are there specific requirements for the *in vivo* use of nanoparticles (for diagnostic or treatment) ? ☐yes ☐no

If yes, please specify, give source/reference and if possible add link:

Are there specific requirements for studies using animal derived products? ☐yes ☐no

If yes, please specify, give source/reference and if possible add link:

Are there requirements for specific populations?

Healthy volunteers? ☐ **yes** ☐ **no**

If yes, please specify, give source/reference and if possible add link:

Vulnerable population: ☐ **yes** ☐ **no**

What are the relevant categories?

- Children ☐
- Elderly ☐
- Pregnant women ☐
- Lactating women ☐
- Unconscious ☐
- Psychiatric disorders ☐
- Dementia ☐
- Prisoners ☐
- Other ☐

If yes, please specify, give source/reference and if possible add link:

Are there specific requirements for emergency condition or critically ill patients? ☐ **yes** ☐ **no**

If yes, please specify, give source/reference and if possible add link:

Is there a waiver of informed consent under emergency condition or critically ill patients? ☐ **yes** ☐ **no**

If yes, please specify, please give source/reference and if possible add link:

Are minority/ ethnicity/ gender taken into account in the national legislation? ☐**yes** ☐**no**

If yes, please specify, give source/reference and if possible add link:

Is there a national volunteer's file for participants in clinical research? ☐**yes** ☐**no**

If yes, specify the rules to enter participants? (please give source/reference and if possible add link):

Are there compensation fees for volunteers/patients participating in clinical research ? ☐**yes** ☐**no**

If yes, under which circumstances, and is there a yearly upper limit? (please give source/reference and if possible add link):

Are there specific strategies for monitoring clinical trials? ☐**yes** ☐**no**

(for example:1-adaptive monitoring based on gradual approach according to the level of risk associated with research, 2- centralised monitoring, 3-monitoring by sampling**)**

If yes, please specify in which type of trial and the strategy used (please give source/reference and if possible add link):

Are there regulatory requirements regarding data management in clinical trials ? ☐**yes** ☐**no**

If yes, please specify for which category of research (please give source/reference and if possible add link):

<u>Are there specific requirements regarding personal data protection in clinical research?</u> ☐**yes** ☐**no**

If yes, please specify under which condition, for which category of research, and the name of the relevant board or authority (please give source/reference and if possible add link):

<u>Are there specific requirements regarding blood / tissue samples (circulation and storage)?</u> ☐**yes** ☐**no**

If yes, please specify, give source/reference and if possible add link:

<u>Are there specific requirements regarding studies on biomarkers/surrogate markers (definition or validation of biomarkers)?</u> ☐**yes** ☐**no**

If yes, please specify, give source/reference and if possible add link:

<u>Are there specific requirements regarding genetic or genotype/phenotype studies?</u> ☐**yes** ☐**no**

If yes, please specify, give source/reference and if possible add link:

Is there a national plan in your country on where to register clinical trials (a register where trial information can be made publicly available before inclusion of the first participant)? ☐**yes** ☐**no**

If yes, please specify, give source/reference and if possible add link:

Is there a national plan on where to register anonymised data from the trial once it has been conducted and analysed? ☐**yes** ☐**no**

If yes, please specify, give source/reference and if possible add link:

Is there a national plan on where to register publications deriving from the clinical trial? ☐**yes** ☐**no**

If yes, please specify, give source/reference and if possible add link:

Is there an obligation to inform the patients on the outcome of the clinical trial? ☐**yes** ☐**no**

If yes, please specify, give source/reference and if possible add link:

Does the legislative system in your country cover any biomedical research? ☐**yes** ☐**no**

or is it focusing on clinical research on health products ? ☐**yes** ☐**no**

Please specify, give source/reference and if possible add link:

Please specify the five top priority topics to improve European clinical research and provide suggestions for improvement:

Problem topic **Suggestions for improvement**

1.

2.

3.

4.

5.

Please specify the five top priority topics to improve European competent authority working practice and provide suggestions for improvement:

Problem topic **Suggestions for improvement**

1.

2.

3.

4.

5.

<u>What would be your expectations regarding future EU regulation on clinical research?</u>

<u>Please indicate who filled out this questionnaire and their phone numbers and e-mails</u>
